# Keratin 86 is up-regulated in the uterus during implantation, induced by oestradiol

**DOI:** 10.1186/s12861-020-0208-6

**Published:** 2020-02-07

**Authors:** He Zhang, Huashan Zhao, Xi Wang, Xiaolin Cui, Lingling Jin

**Affiliations:** 1 College of Basic Medical Sciences, Dalian Medical University, No. 9 West Section Lvshun South Road, Dalian, 116044 Liaoning China; 2 Center for Reproduction and Health Development, Shenzhen Institutes of Advanced Technology, Chinese Academy of Sciences, Shenzhen, 518055 China

**Keywords:** Implantation, Uterus, Oestrogen, Progesterone, Keratin 86

## Abstract

**Background:**

Uterine receptivity is one of the determinants of embryo implantation, which is responsible for pregnancy success. Aberrant embryo implantation due to disrupted uterine receptivity is usually found in ovarian hyperstimulation induced hyperoestrogen patients.

**Results:**

This study identified keratin 86 (KRT86), a fibrous structural protein, which was upregulated in uterine endometrium during peri-implantation. Using a hyperoestrogen mouse model established in a previous study, we found abnormal oestradiol (E2) levels during pre-implantation could trigger high expression of *Krt86* in the uterine epithelium. In an ovariectomised mouse model, combining oestrogen receptors ERα and ERβ knockout mice models, uterine *Krt86* was found to be up-regulated after E2 treatment, mediated by nuclear ERα. Furthermore, we found progesterone (P4) could ameliorate *Krt86* expression, induced by abnormal E2.

**Conclusions:**

These results revealed the dynamic expression and regulation of *Krt86*, especially in hyperoestrogen treated mice, indicating it might act as a marker for non-receptive uterus.

## Background

Embryo implantation establishes pregnancy [1]. During this process, uterine endometrium acquires the ability to accept embryo implantation, which is called uterine receptivity, and is regulated by steroid hormones. Precisely regulated progesterone (P4) and oestrogen (E2) determine uterine receptivity and embryo implantation. In mice, increased ovarian P4 insures uterine receptivity, and then a small surge of oestrogen triggers the initiation of embryo implantation. Disturbances of P4 and E2 during this process can affect implantation, for example, hyperoestrogen induced by ovarian hyperstimulation in in vitro fertilisation (IVF) treatment disrupts embryo implantation. Although different genes have been found to participate in uterine receptivity and embryo implantation regulation, gene changes under the stress of implantation remain less well-known. Given that implantation could be considered a form of xenoimplantation, proteins which regulate epithelial cell sensing and that protect against outside stress may participate in this process. Keratin proteins are a structural component of cells that protect against mechanical stress. Many kinds of keratins exist in adult epithelial cells, for instance, keratin intermediate filaments protect epithelial cells from various stresses that cause cell rupture and death [2]. The study of many human diseases and murine knockout models has testified to its importance as a mechanical stabiliser in epithelia, when integrity is destroyed [3]. Keratin 86 (KRT86), a type II hair keratin, belongs to the keratin gene family, which can form hair and nail structures by heterodimerising with type I keratins [4]. Mutations in *Krt86* are related to a rare hereditary hair loss disorder, monilethrix [5, 6], where hair has a beaded structure, with elliptical nodes and constricted internodes, and is sensitive to damage from weather conditions and fracture [5]. Given the role of KRT86 in ameliorating outside stress, its presence in uterine epithelium might maintain the cytoskeleton, to facilitate embryo implantation.

In the current study, using hyperoestrogen and ovariectomised mouse models, we found that *Krt86* was specifically expressed during embryo implantation, and was up-regulated by E2 through the nuclear oestrogen receptor ERα pathway. In the hyperoestrogen mouse model, which we established in previous study, *Krt86* was found to be up-regulated in the mouse uterus, indicating it might participate in the regulation of uterine receptivity, especially in ovarian hyperstimulation syndrome patients.

## Results

**Fig. 1:**
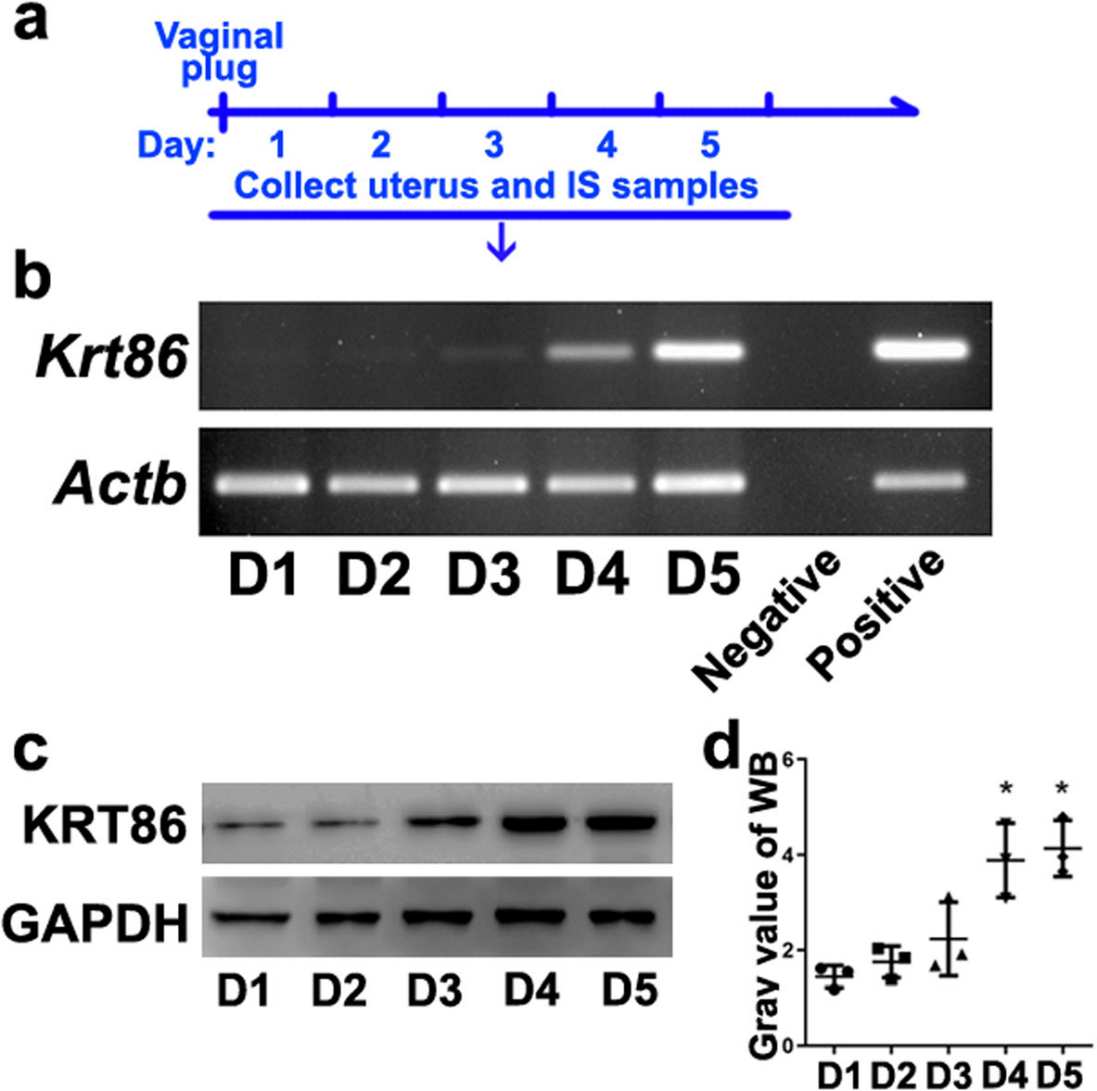
Specific expression of *Krt86* during implantation. **a** Schematic diagram of sample selection in days. IS indicates implantation site. **b** RT-PCR test of Krt86 expression during embryo implantation for days 1–5. **c** KRT86 protein levels assessed by western blot, during implantation days 1–5. **d** Grayscale values of KRT86 protein test. The data is presented as means ± standard deviation (SD), and asterisks indicate statistically significant differences with a *P* value < 0.05

We first examined *Krt86* expression during early pregnancy, for days 1–5 (Fig. 1a). Although low *krt86* was found from day 1 to day 3, on day 4 and day 5 it was significantly up-regulated (Fig. 1b). KRT86 protein changes in the uteri showed a similar pattern as the gene expression results (Fig. 1c, d).

**Fig. 2:**
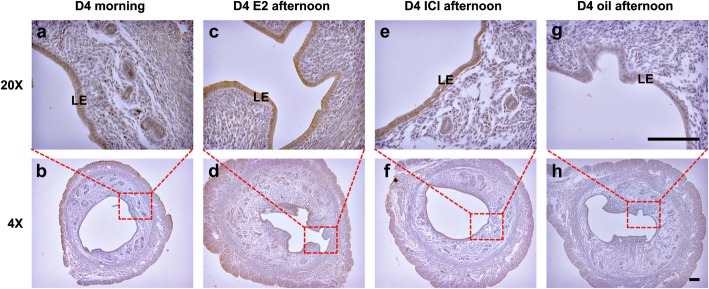
KRT86 is located in the epithelium of the uterus and shows strong expression after treatment with E2. **a**, **b** KRT86 localisation in uterus on morning of day 4 sample. **c**, **d** KRT86 expression on afternoon of day 4, after treatment with E2. **e**, **f** KRT86 expression on day 4 after treatment with oestrogen receptor antagonist ICI 182780. **g**, **h** KRT86 expression for control group after treatment with oil. LE, luminal epithelium. Bar = 500 μm. The staining of KRT86 antibody is brown, the nucleus is blue

Immunohistochemistry analysis for KRT86 protein on the morning of day 4 showed weak expression in the epithelium, compared with the oil treated control group (Fig. 2a, b, g, h). However, enhanced staining of KRT86 was observed after E2 treatment. Notably, KRT86 was specifically localised to the epithelium of uteri (Fig. 2c, d), compared with the oestrogen receptor antagonist ICI 182780 treated group (Fig. 2e, f), suggesting that KRT86 might play a role in hyperoestrogen-induced uteri.

**Fig. 3:**
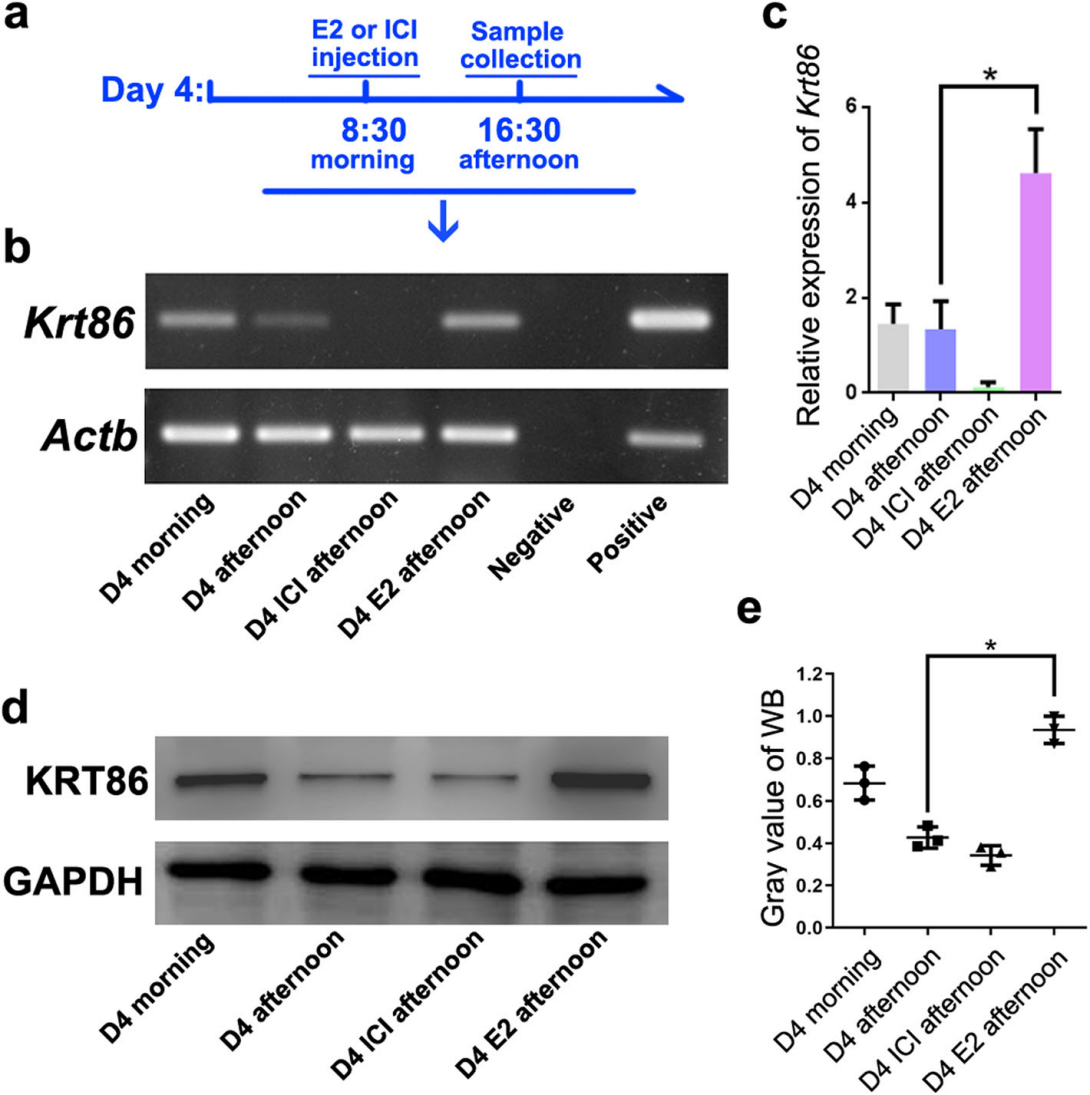
*Krt86* is highly expressed on day 4, after treatment with E2. **a** Schematic diagram of sample collection strategy on day 4. **b** RT-PCR test for *Krt86* at different times for non-treated samples or those treated with oestrogen receptor antagonist ICI 182780 and E2 on day 4. **c** Real-time PCR test for *Krt86* on day 4 after estrogen and oestrogen receptor antagonist ICI 182780 administration. **d** KRT86 protein levels by western blot. **e** Grayscale values of western blot for KRT86 protein levels. The asterisks indicate *P* < 0.05

To verify whether *Krt86* expression was induced by supraphysiological levels of E2 during peri-imlplantation, we treated pregnant mice with E2 (> 50 ng) on the morning of day 4 (8:30) [7], and collect samples at different time (Fig. 3a). Using this hyperoestrogen-induced pathophysiological mouse model, we examined *Krt86* expression levels in day 4 uteri (untreated, treated with E2 and treated with ER antagonist ICI 182780), by RT-PCR and western blotting. Expression of *Krt86* was observed on the morning and afternoon of day 4, with high expression after E2 treatment. For the ER antagonist treated group, *Krt86* was hardly detected; however, when also treatment with E2 *Krt86* had a similar expression to that in mice with E2 alone, on the morning of day 4 (Fig. 3b). Similar results were obtained through real-time PCR (Fig. 3c). Gene expression levels were also reflected by protein analysis, with *Krt86* showing high expression in response to E2 treatment (Fig. 3d, e).

**Fig. 4:**
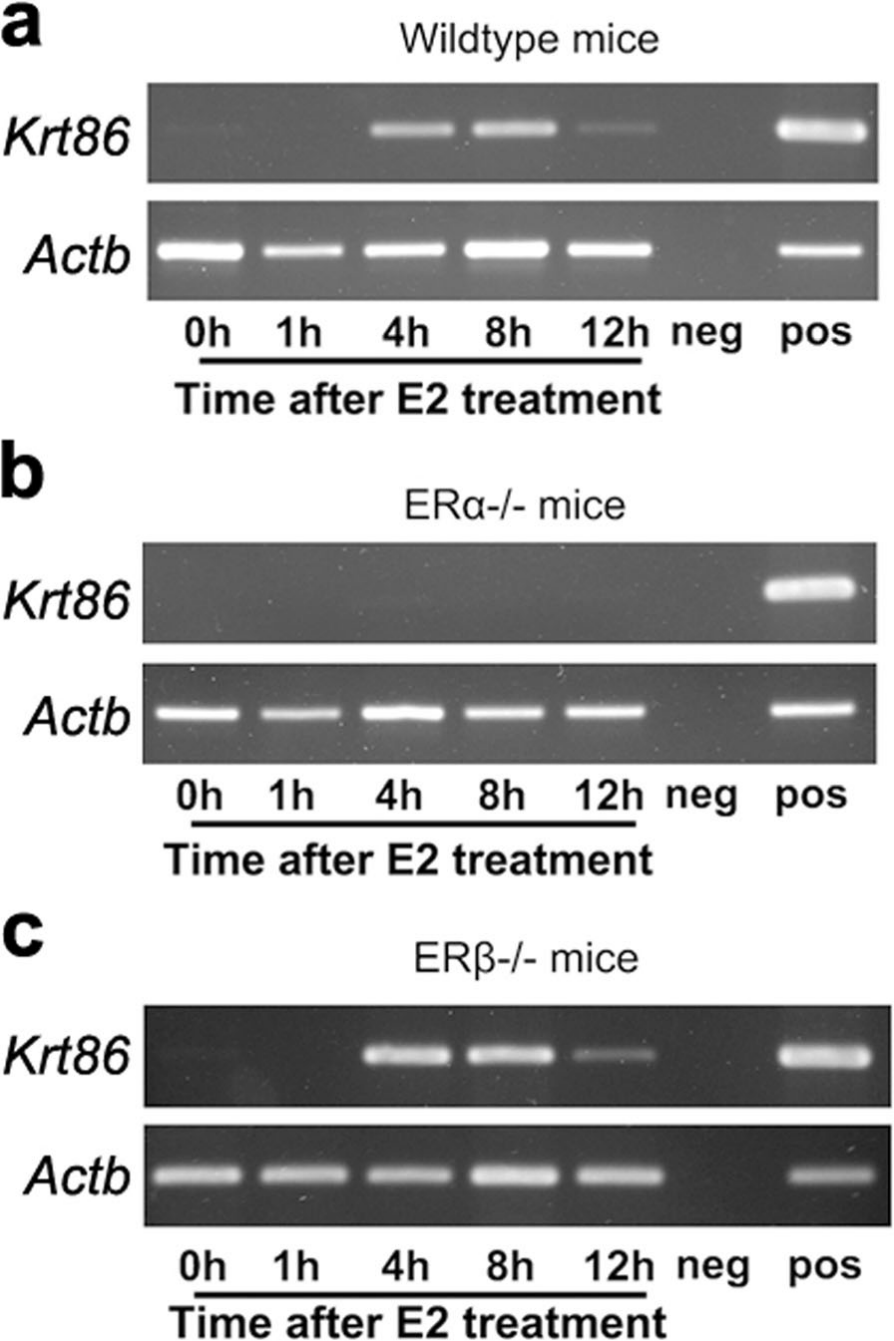
*Krt86* expression at different times after E2 treatment. **a***Krt86* was expressed 4 h after treatment with E2 in wild type (WT) mice. **b***Krt86* not detected in ERα KO mice. **c***Krt86* showed similar expression levels in WT and ERβ KO mice

As ERs were present as two types, ERα and ERβ, we used ERα and ERβ knockout mice combined with an ovariectomised mouse model to identify which one contributed to the high expression of *Krt86*. In wild type (WT) mice, *Krt86* showed transient expression at 4 h and 8 h after E2 treatment, due to an E2-indued affect (Fig. 4a). However, in ERα KO mice, there was no expression of *Krt86*, suggesting that ERα regulated *Krt86* expression (Fig. 4b). By contrast, treating ERβ KO mice with E2 resulted in a similar pattern of *Krt86* expression as the WT group (Fig. 4c).

**Fig. 5:**
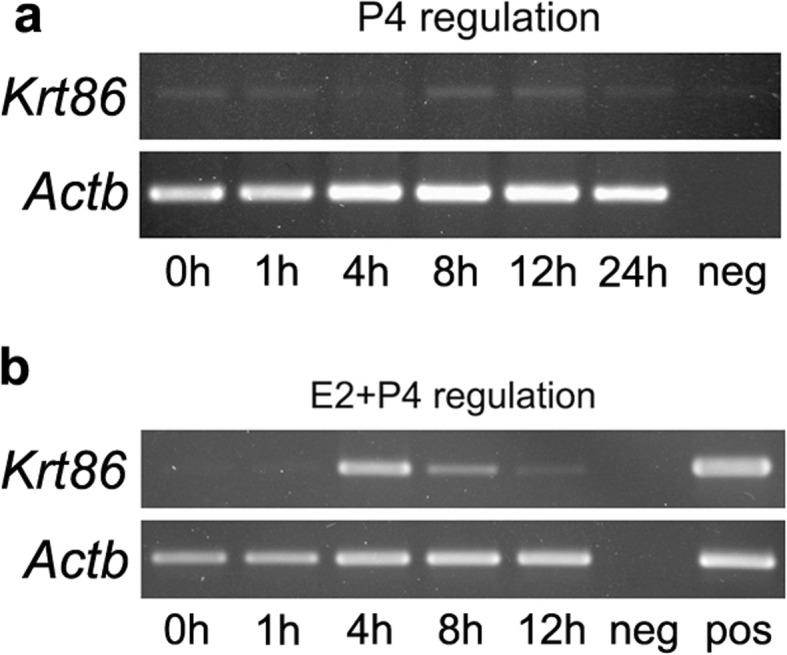
*Krt86* expression test at different times after P4 treatment. **a** RT-PCR test for *Krt86* expression after P4 treatment. **b** RT-PCR test for *Krt86* expression after P4 and E2 treatment

In addition, treatment with only P4 of ovariectomised mice hardly changed *Krt86* expression levels (Fig. 5a). However, expression of *Krt86* weakened at 8 h after co-injection of P4 with E2 (Fig. 5b), suggesting that a hyperoestrogen state up-regulated *Krt86* expression, and P4 treatment antagonised this up-regulation to some extent.

## Discussion

This study demonstrated that uterine KRT86 was dynamically expressed during early pregnancy, and was regulated by oestrogen through ERα signalling. It was also up-regulated in the hyperoestrogen uterus and could be down regulated by P4. This dynamic regulation of KRT86 by E2 suggested that structural changes in the uterus epithelium might be one of reasons for embryo implantation failure in clinical IVF.

Prior to implantation, the endometrial epithelium becomes flattened in order to establish a connection between the embryo and the uterus. Along with embryo migration and invasion in uteri, these mechanical stresses would trigger changes in the structure of the epithelium cytoskeleton. It has been reported that enhanced uterine oestrogen leads to defective luminal epithelium characterised by more epithelial proliferation and abnormal surface transformation [8], suggesting a defective cytoskeleton arrangement in the epithelium. For instance, E-cadherin, a transmembrane adhesion molecule, regulates the cytoskeleton dynamically [9], which is up-regulated in the luminal epithelium before implantation [10], indicating that remodelling of the cytoskeleton in epithelial cells is critical for embryo implantation. E-cadherin is a prerequisite for blastocyst implantation [11]; however, persistent expression of E-cadherin results in implantation failure [12, 13], due to disorganisation of cellular structures. Given that *Krt86* showed dynamically expression in the current study, we presumed *Krt86* might participate in the process of uterine surface transformation. Therefore, further study of *Krt86* in subcellular structures during implantation might reveal more information about the function of KRT86 in the uterus.

The keratin skeleton allow rapid local reconstruction. Keratin filaments are considered to be an important part of the cytoskeleton in epidermal cells. Keratin filaments have high viscoelasticity and flexibility, but it would be hardened with the increase of deformation. It could bend quickly without breaking and recover quickly after withdrawing the deformation pressure [14, 15]. Keratin can reestablish itself depending on the condition change such as salt or PH, which may contribute to the hardness change of keratin. Therefore, we speculated that during embryo implantation KRT86 protein expression increased in the epithelial cells of the uterine cavity, which enhanced the hardness of uterine cavity epithelium, facilitating the embryo implantation. However, under the high estrogen level, the epithelial epithelium KRT86 was overexpressed, which broke the balance that affected embryo attachment and penetration.

There are few reports on the expression and function of KRT86 in the uterus; however, some information can be obtained from homologues. For example, KRT85, another member of the keratin family, is also expressed in the uterus [16]. Like KRT86, it also forms type II hair keratin and nails, and is a basic protein [17]. In a vascular endothelial growth factor (VEGF) regulating implantation model, VEGF repression caused implantation failure, and *Krt85* is dramatically up-regulated (1000 fold), indicating a role of keratin during implantation [16]. In alopecia areata (AA) lesional skin, *Krt85* and *Krt86* were significantly repressed [18]. They are early and middle markers for Alopecia areata, respectively [19, 20]. *Krt86* and *Krt85* have 91% sequence similarity. In Hirosaki hairless rat, *Krt86* and *Krt85* could not be detected [21], suggesting these genes are related with hair follicle development. Based on these reports, we proposed KRT86 was responsible for formation of epithelium of the endometrium, functioning with other keratins during implantation. However, as shown in this current study, high level E2 increased keratin expression, leading to excess keratinisation that might affect implantation.

E2 acts by binding to ERα and ERβ, which are a kind of nuclear steroid hormone receptor [22, 23]. Oestrogen signalling is selectively activated or inhibited based on ERα and ERβ [24]. Using ERα and ERβ knockout mice, the general role of oestrogen signalling has been demonstrated [25, 26]. Male and female ERα knockout mice are infertile, whereas ERβ female mice show inefficient fertility, while ERβ male mice have no apparent defect [24, 27]. Some relevant literature showed that the ERα highly regulates *Krt86* expression after E2 treatment [28, 29]. However, in other cell line models, the ERβ would not affect the *Krt86* expression [30–33]. In addion, E2 is critical for the reproductive organ to achieve pregnancy [34]. Implantation is a complicated process triggered by an E2 surge [35]. Correspondingly, in this current study, ERα was identified as being essential for regulation of *Krt86*, suggesting that it affected the epithelium by modulating keratins. Our data suggested that the KRT86 might be a valuable marker for monitoring IVF and embryo transfer outcomes.

## Conclusions

In summary, the present study showed *Krt86* was highly expressed in the presence of physiological and supraphsiological levels of E2. An aberrant cytoskeleton of the uterus epithelium could be an aspect of implantation failure. Therefore, specific expression of *Krt86* induced by E2 warrants further investigation.

## Methods

### Mice

Female adult ICR mice (7–8 weeks old) were purchased from the Laboratory Animal Centre of Dalian Medical University, China. ERα and ERβ knockout mice were fed in the Laboratory Animal Centre of Dalian Medical University. The Guidelines for the Care and Use of Animals in Research were followed. Care of mice and their handling were conducted in accordance with the Animal Research Committee guidelines of Dalian Medical University. Mice were allowed free access to water and food in 12 h light, 12 h dark conditions. Adult virgin female mice were mated with fertile males at room temperature (25 °C). The morning of finding a vaginal plug was designated as day 1 of pregnancy. Mice were killed at 8:30 a.m. on various days of pregnancy to collect uterine samples, especially for the implantation site (IS). Pregnancy on days 1–4 was confirmed by recovering embryos from the reproductive tracts, and IS on day 5 of pregnancy were identified by intravenous injection of 0.4 ml 0.5% trypan blue in saline 5 min before the mice were killed. Mice were euthanized by dislocating their neck.

### Drug treatment

To induce the supraphysiological E2 mice model, dosage of E2 was as previously described [7]. Corn oil containing 100 ng E2 was administered by subcutaneous injection (s.c.) at 08:30 a.m. on the morning of day 4, with vehicle group mice being injected with oil alone. Some of the day 4 mice were injected with the oestrogen receptor antagonist ICI 182780 (100 μg/mouse) at 08:30 a.m., with uteri being collected on the afternoon of day 4.

To determine the effects of E2 on *Krt86* expression in uteri, mature female ERα and ERβ knockout ICR mice were ovariectomised. After two weeks rest, they were then treat with subcutaneous injections of E2 (Sigma; 100 ng/mouse) dissolved in corn oil (Santa). Uteri from these mice were collected at 0, 1, 4, 8, and 12 h after each treatment, and frozen in liquid nitrogen for further analysis (three mice per time point).

To examine the effects of P4 on the expression of *Krt86* in uteri, ovariectomised mice had s.c. injections of P4 (2 mg/mouse). To neutralise the effect of E2, a co-injection of P4 (2 mg/mouse) was administered along with E2 (100 ng/mouse). Mice were killed, and their uteri were collected at 0, 1, 4, 8, 12 and 24 h after each treatment, and frozen in liquid nitrogen for further analysis (three mice per time point).

### Reverse transcription polymerase chain reaction (RT-PCR) and real-time PCR

Total RNA was isolated from uteri using TRIzol reagent (Invitrogen), according to the manufacturer’s instructions. Total RNA (2 μg) was reverse-transcribed into cDNA using Moloney murine leukemia virus (M-MuLV) reverse transcriptase (M0253, New England BioLabs) and 2 μl Oligo (dT) primers (PC2450, Solarbio). Subsequently, PCR mixes contained 1 μl cDNA, 1 μl specific primers, and 10 μl 2X PCR SuperMix (AS111, TransGen Biotech) in a final volume of 20 μl. The primers used were: *Krt86* forward, 5′-CAGCAGGTTCGCGGCCTTCA-3′; reverse, 5′-GCCTCTGCGTTGGCCTCCAG-3; *Atcb* forward, 5′-TGGAATCCTGTGGCATCCATGAAAC-3′; and *Atcb* reverse, 5′-TAAAACGCAGCTCAGTAACAGTCCG-3. The reaction conditions were as follows: polymerase activation and DNA denaturation (one cycle at 95 °C for 30 s); denaturation, annealing, and extension (30 cycles of 95 °C for 30 s, 60 °C for 30 s and 72 °C for 30 s); and extension (72 °C, 10 min).

Real-time PCR was performed with the Agilent StrataGene Mx3005P QPCR detection system (Agilent StrataGene, USA). cDNA (1 μl) and 0.5 μM primers were adjusted with RNase-free water to a volume of 10 μl, followed by the addition of 10 μL SYBR Green master mix (A6002, Promega). Primers for the detection of *Krt86* were forward, 5′-GAGCAACATGGAGCCTCTGT-3′ and reverse, 5′-CCCGGAGTGCAACTTCTTCT-3, while for *Atcb* they were forward, 5′-TGGAATCCTGTGGCATCCATGAAAC-3′ and reverse, 5′-TAAAACGCAGCTCAGTAACAGTCCG-3′. Reactions took place at 95 °C for 1 min, followed by 40 cycles of 95 °C for 15 s and 60 °C for 1 min. The program for the melting curve analysis was 95 °C for 5 s and 65 °C for 1 min. The mRNA expression levels were quantified using the 2^-△△Ct^ method. Amplification of *Atcb* mRNA was performed to normalise the data. Mouse skin was used as a positive control (pos), while water was used as a negative control (neg).

### Immunostaining

Mouse uteri were cut into small pieces, fixed in Bouin’s solution for 24 h, dehydrated, and embedded in paraffin. Five μm paraffin-embedded endometrial sections were deparaffinised and rehydrated. Endogenous peroxidase activity was blocked by incubating the sections in 3% peroxide in methanol for 10 min at room temperature. After three washes in phosphate-buffered saline (PBS), nonspecific binding was blocked in PBS with 5% rabbit serum for 1 h at room temperature, followed by incubation overnight at 4 °C with 1:100 goat anti-KRT86 primary antibody (sc-168332, Santa Cruz Biotechnology) in blocking solution. After a further three washes in PBS, the sections were incubated for 1 h at 37 °C with 1:100 horseradish peroxidase (HRP)-conjugated rabbit anti-goat IgG (ZB-2306, Zhongshan Biotechnology) in blocking solution, as the secondary antibody. Antibody labelling was detected with fresh diaminobenzidine (DAB) solution (ZLI-9017, Zhongshan Biotechnology), and sections were counterstained with Harris hematoxylin. Photographs of the slides were taken using a Leica microscope.

### Western blot

Proteins were extracted from uterine tissues at various stages after treatment, utilising ice-cold radioimmunoprecipitation assay (RIPA) buffer that was supplemented with protease inhibitor phenylmethylsulfonyl fluoride (PMSF; Beyotime). Protein concentrations were determined using a BCA Protein Assay Kit (Beyotime). Twenty μg protein samples were separated by 12% sodium dodecyl sulphate-polyacrylamide gel electrophoresis and then transferred to polyvinylidene difluoride (PVDF) membranes. The PVDF membranes were blocked with 5% non-fat milk in TRIS-buffered saline at room temperature for 1 h, and then incubated in primary antibodies against KRT86 (Santa Cruz) and beta-actin (1:1000; Beyotime) at 4 °C overnight. After washing three times, the PVDF membranes were probed with secondary antibodies, 1:10,000 HRP-conjugated rabbit anti-goat or goat anti-mouse IgG (Zhongshan Biotechnology), for 1 h at room temperature. Finally, HRP-tagged bands were visualised using the Immobilon western chemiluminescent HRP substrate (WBKLS500, Milipore), and chemiluminescence intensity of each band was quantified by ImageJ software.

### Statistical analysis

Statistical analysis was carried out using the GraphPad 6 program. Each experiment was performed at least three times. One-way analysis of variance, followed by a least-significant-difference test, was used for statistical comparisons among multiple groups. Statistically significant differences were determined at *P* < 0.05.

## Data Availability

All data generated or analysed during this study are included in this published article.

## Abbreviations

AA: Alopecia areata
E2: Oestradiol
IVF: in vitro fertilisation
KRT86: Keratin 86
P4: Progesterone
VEGF: Vascular endothelial growth factor
